# Successful percutaneous retrieval of a detached MitraClip® in the left atrium via maintained gripper line: a case report

**DOI:** 10.1093/ehjcr/ytaf124

**Published:** 2025-03-08

**Authors:** Hiroto Yagasaki, Takeki Suzuki, Ryota Watanabe, Keitaro Watanabe, Toshiyuki Noda

**Affiliations:** Department of Cardiology, Gifu Prefectural General Medical Center, 4-6-1 Noisshiki, Gifu 500-8717, Japan; Department of Medicine, Indiana University School of Medicine, 340 West 10th Street, Indianapolis, IN 46202-3082, USA; Department of Medicine, Indiana University School of Medicine, 340 West 10th Street, Indianapolis, IN 46202-3082, USA; Department of Cardiology, Gifu Prefectural General Medical Center, 4-6-1 Noisshiki, Gifu 500-8717, Japan; Department of Cardiology, Gifu Prefectural General Medical Center, 4-6-1 Noisshiki, Gifu 500-8717, Japan; Department of Cardiology, Gifu Prefectural General Medical Center, 4-6-1 Noisshiki, Gifu 500-8717, Japan

**Keywords:** Transcatheter edge-to-edge mitral valve repair, MitraClip, Mitral regurgitation, Percutaneous retrieval, Case report

## Abstract

**Background:**

Mitral valve transcatheter edge-to-edge repair (M-TEER) with MitraClip® has become an established treatment for high-risk patients with mitral regurgitation (MR). While device-related complications are known, they typically occur during leaflet grasping or after deployment, with most issues arising from device-tissue interaction.

**Case summary:**

A 67-year-old woman with dilated cardiomyopathy underwent M-TEER for severe functional MR. Although resistance was noted during initial device preparation and loading, limited functional testing appeared normal and the procedure was continued. During clip manipulation in the left atrium, mechanical resistance in the arm positioner led to unexpected detachment of the MitraClip, connected only by the gripper line. Through careful traction under echocardiographic and fluoroscopic guidance, we successfully retrieved the clip percutaneously. The procedure was completed with new MitraClip systems, achieving mild residual MR. The patient's symptoms improved from New York Heart Association Classes II and III to I, with sustained improvement during 4.5 years of follow-up.

**Discussion:**

This case reveals a novel mechanism of MitraClip detachment through mechanical failure during preparation, where introducer damage led to harness deformation and subsequent clip detachment. Among available retrieval options, we demonstrated the feasibility of direct traction retrieval under specific conditions, offering a less invasive solution when gripper line connection is maintained. This case emphasizes the importance of meticulous device inspection and preparation, while providing insights into both failure mechanisms and retrieval strategies in M-TEER complications.

**Learning Points**

- Device malfunction may result from introducer damage during preparation, emphasizing the importance of thorough system inspection and functional testing before mitral valve transcatheter edge-to-edge repair procedures.
- When clip detachment occurs with maintained gripper line connection, controlled percutaneous retrieval under continuous imaging guidance represents a feasible rescue strategy.

## Introduction

Mitral valve transcatheter edge-to-edge repair (M-TEER) with MitraClip® (Abbott Vascular, Santa Clara, CA, USA) has become an established treatment for high-risk patients with mitral regurgitation (MR).^1,2^ Complications such as clip detachment and embolization can occur during leaflet grasping or after device release.^3–8^ We present the first documented case of MitraClip detachment in the left atrium (LA) with maintained gripper line connection, emphasizing critical aspects of device preparation.

## Summary figure

**Figure:**
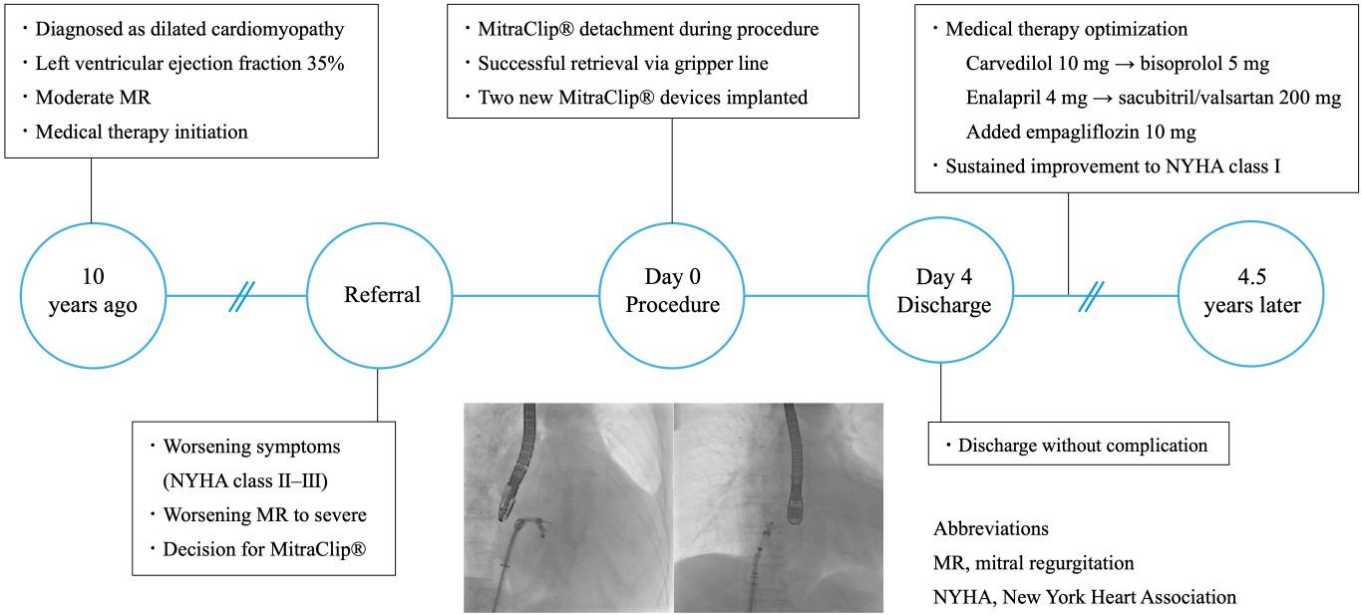


## Case presentation

A 67-year-old woman with dilated cardiomyopathy, diagnosed 10 years ago with left ventricular (LV) ejection fraction of 35% (reference range: 50%–70%) and moderate MR, was referred for New York Heart Association (NYHA) Classes II and III dyspnoea. She had no significant cardiovascular risk factors or other comorbidities that would affect the treatment strategy. Her symptoms persisted despite optimal medical therapy with carvedilol 10 mg, enalapril 2.5 mg, spironolactone 25 mg, azosemide 30 mg, and tolvaptan 3.75 mg daily. On physical examination, the patient was 160 cm tall, weighed 57 kg, blood pressure of 123/71 mmHg, and heart rate of 100/min. Cardiac examination revealed a Levine Grade II/VI systolic ejection murmur at the left third intercostal space and mild lower extremity oedema.

**Figure 1:**
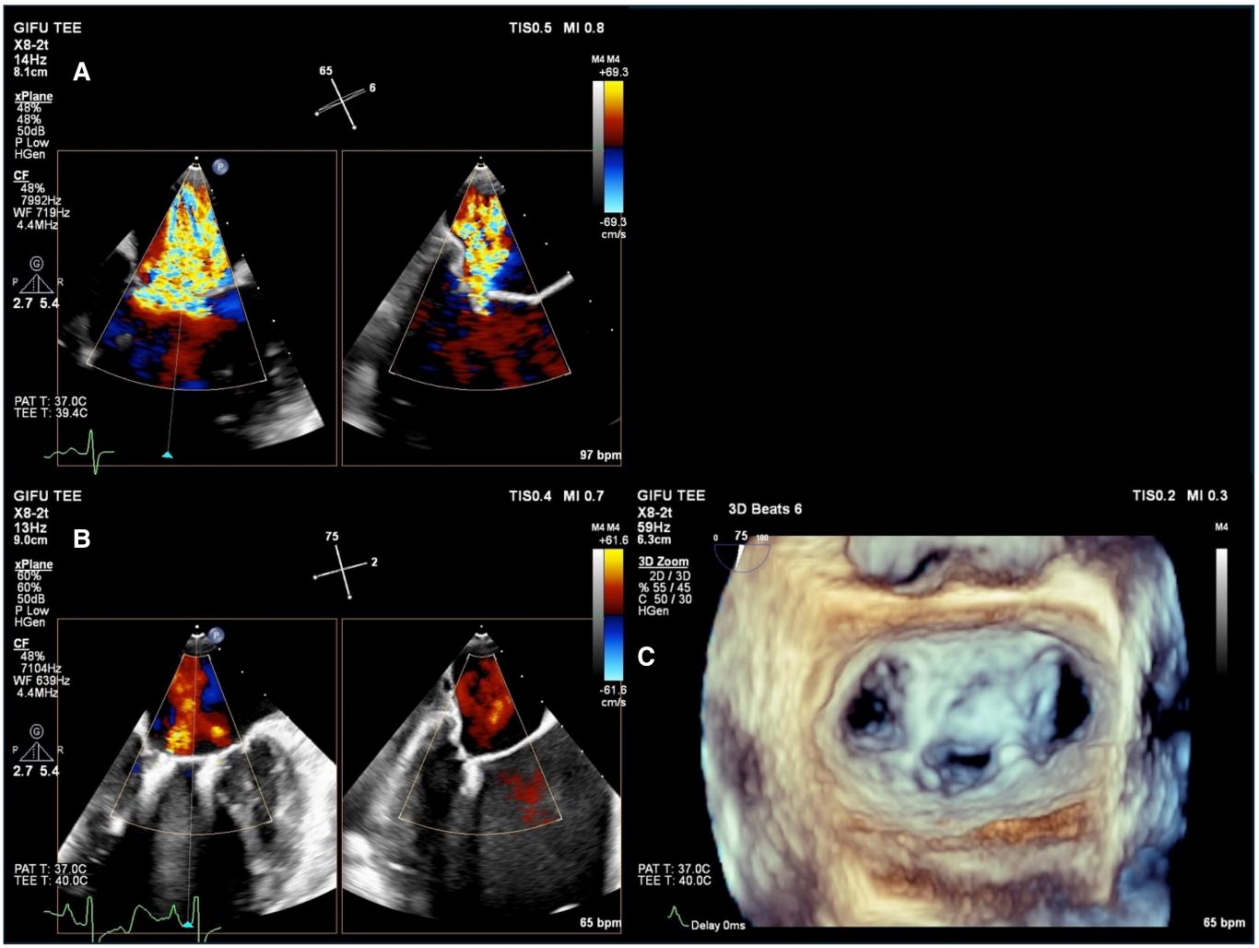
Transoesophageal echocardiography (TEE) imaging. (*A*) Pre-operative transoesophageal echocardiography imaging. (*B*) Post-procedural 2D transoesophageal echocardiography image demonstrating clip position. (*C*) Post-procedural 3D transoesophageal echocardiography image confirming final result.

Outpatient B-type natriuretic peptide levels ranged from 150 to 250 pg/mL (normal range: < 125 pg/mL). Transthoracic echocardiography revealed LV end-diastolic diameter of 70 mm (reference range: 35–52 mm), LV ejection fraction of 31%, and severe functional MR between A2 and P2 scallops, characterized by a regurgitant fraction of 59% (threshold for severe MR: 50%) and effective regurgitant orifice area of 0.27 cm² (threshold for severe MR: 0.20 cm^2^). Transoesophageal echocardiography (TEE) confirmed significant tethering with vena contracta width >11 mm (threshold for severe MR: 7 mm) (*Figure 1A*). Despite a low Society of Thoracic Surgeons score (1.89%), our multidisciplinary team chose M-TEER using two second-generation MitraClip devices considering the LV dysfunction, the functional aetiology of MR, and patient preference for early mobilization.

Mitral valve transcatheter edge-to-edge repair was performed under general anaesthesia. During clip preparation, slight resistance was noted when the clip introducer was sliding over the clip. Although the clip appeared to function normally during checks, the introducer was not thoroughly inspected. The clip was then advanced through the system without difficulty. Following transseptal puncture and advancement to the straddle position, the clip was positioned over the mitral valve without issues. However, when the lock lever was unlocked for opening in the LA, the clip showed restricted movement and would not open beyond 30°.

**Figure 2:**
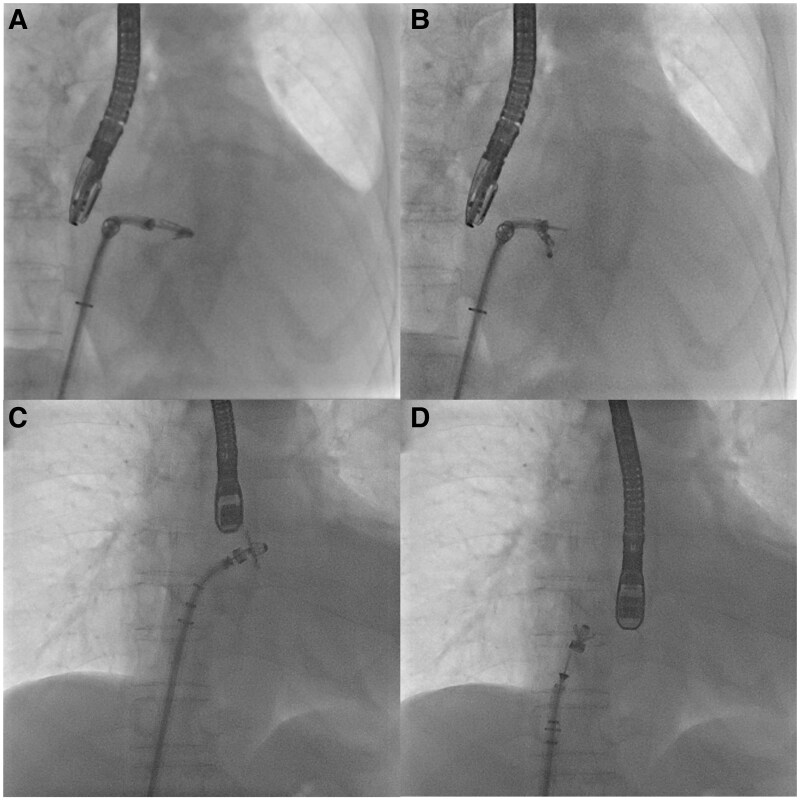
Fluoroscopic sequence of the clip detachment from the clip delivery system (CDS). (*A*) Clip attached to the clip delivery system. (*B*) Complete clip detachment. (*C*) Clip retraction toward the steerable guide catheter. (*D*) Inverted clip position within the right atrium.

Initial attempts to close and retrieve the clip were unsuccessful due to resistance in the arm positioner. As troubleshooting, we returned the arm positioner to neutral and pulled back the lock lever ∼5 mm. We then rotated the arm positioner slightly in the closing direction, followed by rotation in the opening direction. When this manoeuvre failed, we repeated the process with a stronger rotation in the closing direction, which generated unprecedented mechanical resistance. During subsequent rotation in the opening direction, the clip suddenly opened to 180° and detached from the clip delivery system (CDS), remaining connected only by the gripper line in its default raised position (*Figure 2A* and *B*, and Supplementary material online, *Video S1*).

**Figure 3:**
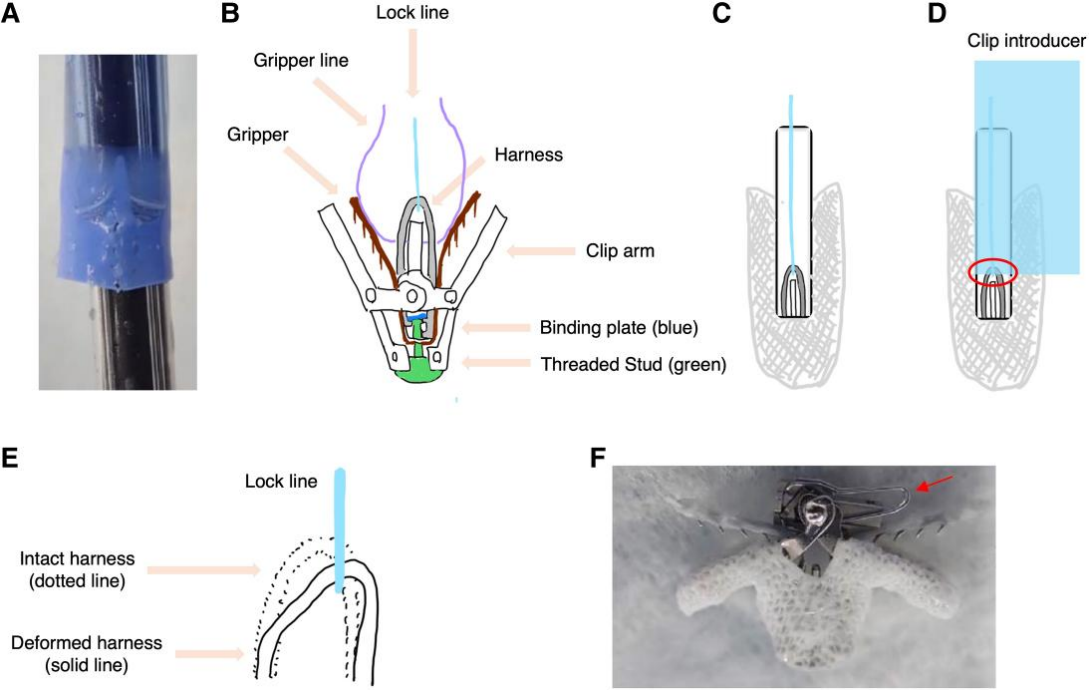
Post-retrieval analysis of the introducer and clip components. (*A*) Introducer tear documentation. (*B*) Structural anatomy and nomenclature of the clip system. (*C*) Clip in closed position. (*D*) Schematic showing introducer sheath entrapment between clip arm and shaft (circle indicates potential harness damage site). (*E*) Schematic of the deformed harness. (*F*) Retrieved harness showing deformation (arrow).

Under TEE and fluoroscopic guidance, we performed direct retrieval of the clip. Although the grippers temporarily displaced downward during detachment, they returned to their raised position during controlled traction toward the steerable guide catheter (SGC). The gripper position was maintained to ensure secure connection. Using gentle and steady traction on the CDS, the clip was gradually guided toward the SGC. The clip inverted during this process and partially entered the SGC, allowing safe withdrawal of the entire system into the right atrium (*Figure 2C* and *D*, and Supplementary material online, *Video S2*). Transoesophageal echocardiography revealed a 2–3 mm atrial septal defect but no other mechanical complications were observed with stable haemodynamics. The CDS and SGC were retrieved via femoral cut-down. Careful inspection of the retrieved system revealed a tear in the introducer that was not visible during the initial preparation (*Figure 3A*).

Following the retrieval of the system, two MitraClip NT devices were successfully implanted using new CDSs and SGC, resulting in mild residual MR (*Figure 1B* and *C*). X-ray and TEE confirmed no device components remained in the patient.

The patient was discharged on Day 4 without complications. During outpatient follow-up, pharmacotherapy was optimized: beta-blocker therapy was changed from carvedilol 10 mg to bisoprolol 3.75 mg daily, which was later uptitrated to 5 mg. The angiotensin-converting enzyme inhibitor was switched from enalapril 2.5 mg to perindopril 4 mg daily, and subsequently replaced with sacubitril/valsartan 200 mg daily. Empagliflozin 10 mg daily was added to the regimen following its regulatory approval. Her symptoms had improved to NYHA Class I and remained stable for 4.5 years.

## Discussion

Device-related complications during M-TEER procedures occur in 1%–5% of cases, with MitraClip detachment typically occurring during leaflet grasping or post-deployment.^3,4^ This report analyses a unique case of pre-grasping clip detachment and its direct retrieval via an intact gripper line.

The introducer tear indicated a handling-related complication rather than a manufacturing defect. The location and pattern of the introducer tear suggested entrapment between the clip arm and shaft during insertion (*Figure 3A–D*). This entrapment likely caused mechanical stress on the device system, resulting in harness deformation (*Figure 3E* and *F*).

**Figure 4:**
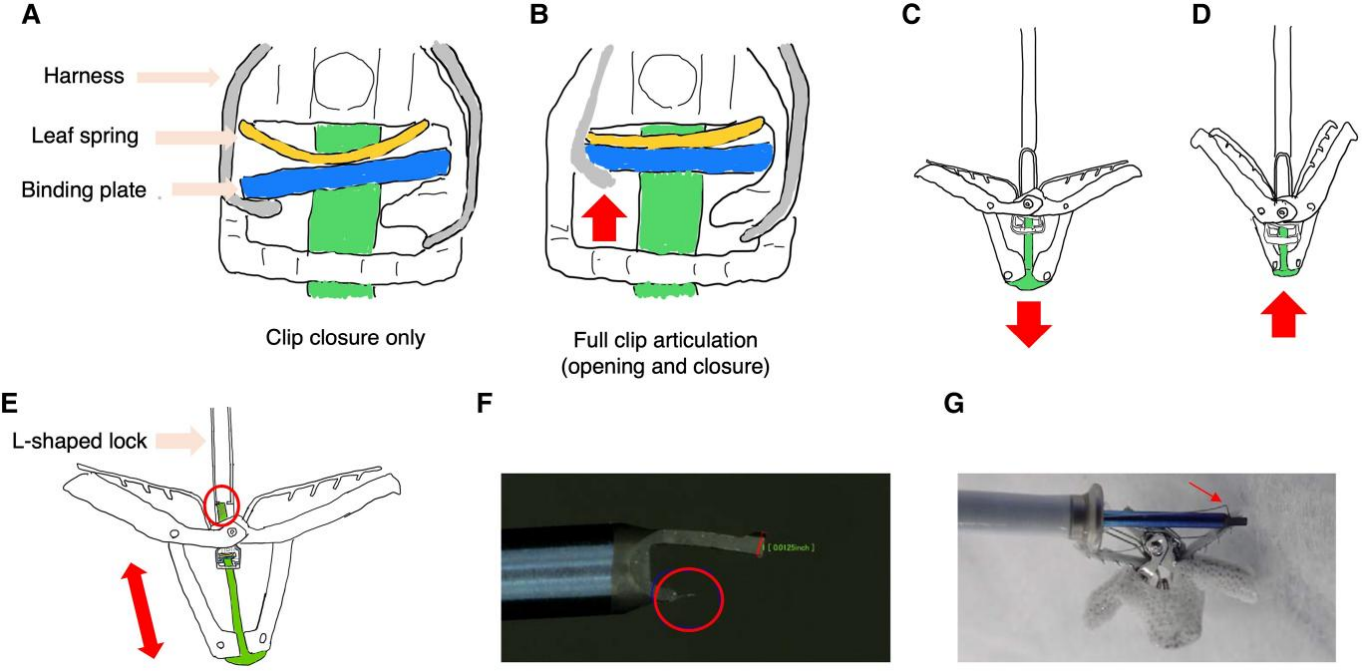
Proposed mechanism of clip failure and structural analysis. (*A*) Normal locked configuration showing lowered lock lever and binding plate. (*B*) Unlocked configuration with elevated lock lever and binding plate (arrow). (*C*) Normal opening mechanism: forward stud advancement (arrow) via arm positioner rotation. (*D*) Normal closing mechanism: backward stud retraction (arrow) via arm positioner rotation. (*E*) Proposed failure mechanism demonstrating shear stress on the L-shaped lock and stud tip (circle) due to repetitive misaligned axial movement (arrow). (*F*) Retrieved device with missing L-shaped lock (circle). (*G*) Retrieved gripper line showing kink pattern (arrow).

Under normal conditions, the harness, which connects to the lock line, controls the binding plate position. The binding plate is typically angled, securing the threaded stud’s position (*Figure 4A*). When the harness is elevated, it raises the leaf spring, causing the binding plate to align horizontally. This alignment positions the plate’s hole with the stud axis, enabling stud movement (*Figure 4B*). This stud movement is synchronized with the arm positioner: rotating it open moves the stud downward to open the clip, while rotating it closed moves the stud upward to close the clip (*Figure 4C* and *D*).

**Figure 5:**
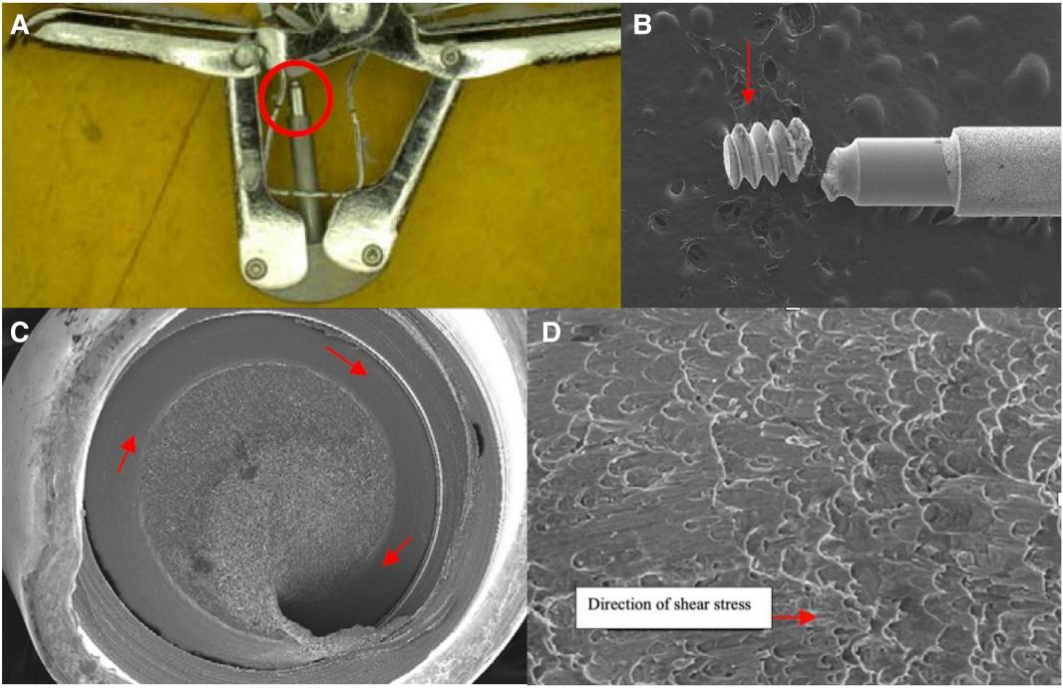
Structural analysis of the fractured threaded stud. (*A*) Stud fracture site (circle). (*B*) Fractured stud segment (arrow). (*C*) Shear stress-induced surface damage (arrow). (*D*) Fracture surface morphology showing shear stress patterns (arrow).

In our case, the deformed harness compromised force transmission through the lock line, resulting in insufficient horizontal positioning of the binding plate. When we first rotated the arm positioner open, the stud advanced along this misaligned axis. Our subsequent attempt to close the clip forced the stud to retract along the same misaligned path, preventing complete clip closure. This misalignment caused excessive stress on one side of the L-shaped lock, potentially damaging it (*Figure 4E* and *F*). The final rotation in the opening direction then applied additional force to the misaligned stud, leading to stud fracture (*Figures 4E* and *5A–D*) and clip detachment, with only the gripper line remaining connected. The kinked state of the gripper line likely occurred during the subsequent retrieval process (*Figure 4G*).

When a MitraClip detaches, various retrieval methods have been reported, including surgical extraction,^5^ snare techniques,^6,7^ and retrograde approaches.^8^ In our case, we chose direct traction retrieval via the gripper line based on three favourable conditions: maintained gripper line connection, stable patient haemodynamics, and optimal clip position in the left atrium. This approach carried risks, particularly clip dislodgement into the left ventricle or mitral leaflet trauma. To minimize these risks, we employed continuous TEE and fluoroscopic guidance during the controlled retrieval process. While our method proved successful, alternative approaches may be necessary in different scenarios. Surgical extraction provides direct access but increases operative risk in high-risk patients. Snare techniques and retrograde approaches, while effective for completely detached clips, may increase procedural complexity and the risk of structural damage.

This experience highlights two preventive measures: meticulous device preparation and thorough functionality testing, with device replacement warranted whenever mechanical concerns arise—despite potential cost implications. Moreover, this case advances our understanding of M-TEER complications through detailed analysis of pre-grasping mechanical failure and successful validation of direct traction retrieval with maintained gripper line connection. Systematic documentation of such rare events will continue to improve procedural safety and outcomes.

## Supplementary Material

ytaf124_Supplementary_Data

## Data Availability

The data underlying this article are available in the article.

## Lead author biography

Dr Hiroto Yagasaki is a cardiologist at Gifu Prefectural General Medical Center, Japan, and a visiting researcher at Indiana University School of Medicine, USA. He specializes in echocardiography and cardiac magnetic resonance imaging, focusing on structural heart disease interventions. At his institution in Japan, he leads procedures including transcatheter aortic valve implantation, edge-to-edge valve repair, left atrial appendage closure, and patent foramen ovale closure. He holds board certification in internal medicine, cardiology, echocardiography, and other cardiovascular subspecialties in Japan.

## Notes

**Consent:** The patient reviewed and provided written informed consent for publication in compliance with COPE guidelines. The consent form is stored at our institution.

## Notes

**Funding:** This research received no specific grant from public, commercial, or not-for-profit funding agencies.
